# Viral Delivery of dsRNA for Control of Insect Agricultural Pests and Vectors of Human Disease: Prospects and Challenges

**DOI:** 10.3389/fphys.2017.00399

**Published:** 2017-06-14

**Authors:** Anna Kolliopoulou, Clauvis N. T. Taning, Guy Smagghe, Luc Swevers

**Affiliations:** ^1^Insect Molecular Genetics and Biotechnology Research Group, Institute of Biosciences and Applications, NCSR “Demokritos,”Aghia Paraskevi, Greece; ^2^Laboratory of Agrozoology, Department of Crop Protection, Faculty of Bioscience Engineering, Ghent UniversityGhent, Belgium

**Keywords:** pest control, recombinant virus, insect-specific virus, arbovirus, plant virus, virus-like particle, RNAi, biosafety

## Abstract

RNAi is applied as a new and safe method for pest control in agriculture but efficiency and specificity of delivery of dsRNA trigger remains a critical issue. Various agents have been proposed to augment dsRNA delivery, such as engineered micro-organisms and synthetic nanoparticles, but the use of viruses has received relatively little attention. Here we present a critical view of the potential of the use of recombinant viruses for efficient and specific delivery of dsRNA. First of all, it requires the availability of plasmid-based reverse genetics systems for virus production, of which an overview is presented. For RNA viruses, their application seems to be straightforward since dsRNA is produced as an intermediate molecule during viral replication, but DNA viruses also have potential through the production of RNA hairpins after transcription. However, application of recombinant virus for dsRNA delivery may not be straightforward in many cases, since viruses can encode RNAi suppressors, and virus-induced silencing effects can be determined by the properties of the encoded RNAi suppressor. An alternative is virus-like particles that retain the efficiency and specificity determinants of natural virions but have encapsidated non-replicating RNA. Finally, the use of viruses raises important safety issues which need to be addressed before application can proceed.

## Introduction

RNA interference (RNAi) is a transforming technology that triggers specific gene silencing through delivery of homologous dsRNA fragments (Mello and Conte, 2004). The technique incited great interest for potential applications in medicine (RNAi-based drugs) (Bouchie, 2014; Haussecker and Kay, 2015) but is also harnessed as a new method for specific and safe pest control in agriculture (Swevers and Smagghe, 2012; Scott et al., 2013; Santos et al., 2014; Nandety et al., 2015; Andrade and Hunter, 2016; Joga et al., 2016; Perkin et al., 2016). In agriculture, RNAi technology has matured such that the first RNAi-based pest control agent, consisting of a transgenic corn that produces dsRNA targeting an essential gene and two Bt toxins for control of the western corn rootworm, was approved for release in the field and commercialization (Hitchon et al., 2016; Joga et al., 2016).

While RNAi-based control of particular coleopteran pests, such as the western corn rootworm *Diabrotica virgifera* (Baum and Roberts, 2014) and the Colorado potato beetle *Leptinotarsa decemlineata* (Palli, 2014), is very feasible because of the high sensitivity of these species to dsRNA in the environment, it is equally clear that significant challenges remain for control of the majority of the other agricultural pests. Recent studies have established that stability and efficient cellular uptake of dsRNA are of utmost importance for effective gene silencing (Christiaens et al., 2014; Ivashuta et al., 2015; Shukla et al., 2016; Wang et al., 2016). While the most straightforward and effective method for delivery of dsRNA to the insect is through feeding, it is well documented that insects secrete dsRNA-degrading enzymes in their gut content (Liu et al., 2013; Wynant et al., 2014). Furthermore, the high pH content in the gut of lepidopteran insects is thought to destabilize unprotected dsRNA. In a comparative study, a much higher stability and tissue penetrance of dsRNA was found for the RNAi-sensitive coleopteran insects than for the RNAi-refractory lepidopterans (Ivashuta et al., 2015). Furthermore, while cell lines from both insect orders are capable of uptake of fluorescent dsRNA, endosomal escape, and interaction with the RNAi machinery in the cytoplasm were only observed in coleopteran lines (Shukla et al., 2016). In a third recent study, a negative correlation could also be established between RNAi efficiency and dsRNA degradation (Wang et al., 2016). From the beginning of the discovery of the RNAi process it has become abundantly clear that dsRNA delivery is the key event and that engineering of sophisticated dsRNA vehicles may be required to trigger gene silencing in more recalcitrant insect species. Once dsRNA is delivered in the cytoplasm, the cellular process of RNAi is very efficient, and robust silencing is triggered even at low doses of dsRNA (Yamaguchi et al., 2011; Kolliopoulou and Swevers, 2013).

For improving the delivery of dsRNA to insects by feeding, two different approaches have been used that are either based on synthetic nanoparticles or employ engineered micro-organisms that produce dsRNA molecules. Regarding synthetic nanoparticles, it was demonstrated that incorporation of dsRNA into chitosan, a natural biodegradable polymer that can be prepared cheaply by deacetylation of chitin, resulted in effective induction of RNAi in mosquitoes by feeding (Zhang et al., 2010; Zhang X. et al., 2015). Other successful encapsulations for dsRNA delivery include liposomes (Whyard et al., 2009; Taning et al., 2016) and carbon quantum dots (Das et al., 2015). For efficient delivery in mammals, small interfering RNAs (siRNAs) are usually chemically modified to increase stability and uptake (Rossi, 2009; Kanasty et al., 2013), but this approach has been used only sparingly in insects (Bryant et al., 2010).

Micro-organisms can be engineered efficiently with appropriate vectors to produce specific dsRNAs and have been employed for delivery of insecticidal dsRNAs. Bacteria-mediated delivery of dsRNA was pioneered in *Caenorhabditis elegans* using the RNase III-deficient *Escherichia coli* strain HT115(DE3) (Kamath and Ahringer, 2003) and this technique was also applied to successfully induce RNAi of target genes in insects (Tian et al., 2009; Zhu et al., 2011). *E. coli* however can act as a pathogen in insects and it would be therefore safer to use natural symbionts of the targeted insects as delivery vectors (Whitten et al., 2016). Similarly, genetically modified algae and yeast have been used to deliver dsRNA in mosquitoes and *Drosophila suzukii*, respectively (Kumar et al., 2013; Murphy et al., 2016).

Plant-mediated RNAi involves the generation of transgenic plants that produce dsRNAs targeting specific insect pests and was an approach that sparked much interest from the beginning (Baum et al., 2007; Mao et al., 2007). However, genetically modified plants expressing dsRNAs take many years to develop and millions of dollars to commercialize due to strict rules from regulatory agencies (Palli, 2014; Andrade and Hunter, 2016). Furthermore, the absence of reliable methods for transformation of many crops (Altpeter et al., 2016) and the general lack of public acceptance toward genetically modified crops are currently significant roadblocks that delay or even prevent applications of plant-mediated RNAi.

When dsRNA is produced in micro-organisms or plants, there is a risk of degradation by endogenous nucleases or processing by the RNAi machinery to small RNAs that are less effective. This was dramatically illustrated in potato plants, where accumulation of dsRNA in chloroplasts was much higher than in the cytoplasm of the plant cells and resulted in much more efficient RNAi effects in targeted Colorado potato beetles (Zhang J. et al., 2015). The high accumulation of dsRNA in plastids is caused by the absence of dsRNA processing machinery in these organelles (Whyard, 2015). Thus, protection in organelles or encapsulation may be necessary for stabilization of dsRNA and its effective transfer to target organisms.

In contrast to the use of bacteria for dsRNA delivery to insect pests, the possible application of engineered viruses for this purpose has received much less attention. Nevertheless, viruses have several attractive properties that make them excellent delivery vehicles for nucleic acids such as efficiency and specificity of infection and the evolved avoidance of the immune response. In mammals, several types of viruses, such as adenovirus, adeno-associated virus, retrovirus, lentivirus, and herpesvirus were successfully engineered as nucleic acid delivery vehicles with their application in gene therapy (Robbins et al., 1998; Wang and Gao, 2014). Virus-based delivery systems are in general considered more efficient than non-viral methods, although the latter are regarded as safer. Given the prevalence of virus-based gene transduction in mammalian models, the paucity of reports using viruses for delivery of dsRNA in insects may seem surprising. This review will focus on the prospects and challenges of using viruses or virus-derived proteins to deliver dsRNA to insect pests in agriculture and vectors of human disease.

## Types of virus infections in insects and the interaction with the RNAi machinery

Viruses that infect insects can be divided into different classes and the type of virus infection will likely determine its suitability to be used as a vector for dsRNA delivery. First, a distinction needs to be made between DNA viruses and RNA viruses. An obvious distinction between these types of viruses is that dsRNA is produced during DNA virus infection only after transcription while RNA viruses produce dsRNA triggers during both replication and transcription. In addition, DNA virus replication and transcription generally occurs in the nucleus, in contrast to the more common cytoplasmic residence of RNA viruses. Because dsRNA is an important trigger of the innate immune response, which includes RNAi in insects (Ding, 2010; Xu and Cherry, 2014; Marques and Imler, 2016), RNA viruses often encode potent viral suppressors of RNAi (VSR) as a defense mechanism (Kingsolver et al., 2013; Gammon and Mello, 2015; Cappelle et al., 2016). Thus, while RNA viruses may at first instance be more suitable to act as RNAi triggers as DNA viruses, this co-evolved defense mechanism should be taken into account in their assessment as dsRNA-delivery vehicles. In the following, a short overview is presented of the viruses that infect insects and their interaction with the RNAi machinery during infection.

### DNA viruses

The best known insect viruses with double-stranded DNA genome are baculoviruses (Baculoviridae) of which ~600 members are described that mainly infect Lepidoptera, but also Hymenoptera and Diptera (Hemiou et al., 2005). The prototype baculovirus species is *Autographa californica* multiple nucleopolyhedrovirus (AcMNPV), of which the infection cycle has been studied in great detail, including transcriptome analysis (Chen et al., 2013; Chen Y.-R. et al., 2014). Despite being a DNA virus, however, a large number of viral siRNAs (vsRNAs), with Dicer-processing signature, can be identified during AcMNPV infection (Mehrabadi et al., 2015). Further evidence, that RNAi is involved in the innate immune response against AcMNPV infection, was the identification of a VSR gene, *p35*, in its genome. Interestingly, p35 was first identified as an inhibitor of apoptosis, but its anti-apoptotic activity was not linked to its capacity to inhibit RNAi (Mehrabadi et al., 2015). A similar occurrence of vsRNAs located to hot spots in the genome was also observed during *Helicoverpa armigera* single NPV (HaSNPV) infections (Jayachandran et al., 2012). Silencing of *Dicer-2* (siRNA pathway), but not *Dicer-1* (miRNA pathway) resulted in higher transcript accumulation and higher viral replication levels. On the other hand, an RNAi strategy was successfully employed to inhibit *Bombyx mori* NPV (BmNPV) replication in transgenic silkworm cells (Kanginakudru et al., 2007; Subbaiah et al., 2013; Zhang P. et al., 2014), indicating that high levels of dsRNAs targeting different essential viral genes could overcome the counter defense mechanisms of the virus.

While vsRNAs are generated during baculovirus infection, presumably after annealing of overlapping complementary transcripts (Jayachandran et al., 2012), several other studies have indicated a role for miRNAs to regulate the baculoviral infection cycle (Kolliopoulou and Swevers, 2014 and references therein). Several miRNA precursors are predicted in the BmNPV genome, of which bmnpv-miR-1 targets the 3′-UTR of the mRNA of the Ran GTP-binding protein, a regulator of the nucleocytoplasmic transport of small RNAs (Singh et al., 2010, 2012). This results in depression of the cellular host miRNA population, including bmo-miR-8, which has multiple binding sites on the mRNA of the viral gene *ie-1*, a master regulator of the viral infection cycle (Singh et al., 2012). While this can be viewed as a clear defense mechanism, baculoviruses can also escape the immune response in more subtle ways by auto-regulating gene expression during the infection cycle. Another miRNA produced by BmNPV, bmnpv-miR-3, was shown to negatively auto-regulate expression of viral late genes to decrease the viral load and the detection by the immune response (Singh et al., 2014). Down-regulation of ODV-E25 mRNA expression by AcMNPV-miR-1 is proposed to accelerate the formation of occlusion-derived virions during the infection cycle (Zhu et al., 2013). Also host cell miRNAs can play a role in the regulation of the baculoviral infection cycle or antiviral defense. During AcMNPV infection, most miRNAs from the host are down-regulated (Mehrabadi et al., 2013), although exceptions exist, such as *bantam*, which is upregulated and negatively regulates viral gene expression and replication (Shi et al., 2016). Finally, it was shown that the miRNA pathway could be harnessed to control BmNPV infection using expression constructs of artificial miRNA precursors (Zhang J. et al., 2014).

Similar interactions with the RNAi machinery were reported for other viruses with large DNA genomes such as ascoviruses and nudiviruses. The infection cycle of *Heliothis virescens ascovirus* (HvAV) is regulated by both cellular and viral miRNAs that target the viral DNA polymerase (Hussain et al., 2008; Hussain and Asgari, 2010). HvAV also encodes an RNase III gene that functions as an inhibitor of gene silencing (Hussain et al., 2010). The miRNA pathway was also shown to be involved in the establishment of latent infections of *Heliothis zea* nudivirus 1 (HzNV-1) (Wu et al., 2011). Another virus with a large DNA genome is the Invertebrate iridescent virus 6 (IIV-6; Iridoviridae) which has a broad host range. IIV-6 is a target of the RNAi machinery in *Drosophila* (Bronkhorst et al., 2012) and produces a dsRNA-binding protein that acts as a VSR during infection (Bronkhorst et al., 2014).

Another group of DNA viruses studied in some detail in insects consists of the densonucleosis viruses, or densoviruses (DNVs). Densoviruses belong to the subfamily Densovirinae within the family Parvoviridae and are characterized by a linear, single-stranded DNA genome of 4–6 kb that is packaged in icosahedral, non-enveloped particles (Bergoin and Tijssen, 2000). Small RNAs corresponding to densoviruses were reported in small RNA deep sequencing data of mosquitoes, but it is unclear whether these are generated by a canonical RNAi pathway (Ma et al., 2011). In DNA viruses, complementary transcripts can anneal to form dsRNA (Bronkhorst et al., 2012), but there do not seem to be overlapping complementary transcripts in densoviruses (Carlson et al., 2006).

The plant-infecting Geminiviridae have single-stranded circular DNA genomes, but have been shown to be susceptible to RNAi in transgenic plants, at least for the genus Begomovirus (Bonfim et al., 2007). Begomoviruses are transmitted by whiteflies in a persistent, circulative manner although it is not clear whether they replicate in insect tissues (Rosen et al., 2015). Reverse genetics systems for begomoviruses are available in plants (Tuttle et al., 2008), thus offering possibilities of engineering for dsRNA delivery in insects, analogous to the engineering of plant RNA virus vectors (see also on Section Plant Viruses).

Viral siRNAs are also produced during infection of *Drosophila* cells with vaccinia virus, a mammalian dsDNA virus that replicates in the cytoplasm (Poxviridae) (Sabin et al., 2013). The most abundant siRNAs are produced from long, structured RNAs derived from the terminal repeats. Interestingly, vaccinia virus infection in *Drosophila cells* results in poly-adenylation and degradation of cellular miRNAs by the viral poly(A) polymerase (Backes et al., 2012). Degradation of miRNAs during infection with entomopoxvirus is also observed in lepidopteran cells (Backes et al., 2012).

### RNA viruses

Insects can also be infected with a wide range of RNA viruses that possess positive-strand single-stranded RNA [(+)ssRNA], negative-strand ssRNA [(−)ssRNA] or double-stranded RNA (dsRNA) genomes. Moreover, the genomes can consist of one or multiple segments (Karlikow et al., 2014; Xu and Cherry, 2014). Many plant RNA viruses rely on insects (mainly Hemiptera) for transmission (Fereres and Raccah, 2015) while mosquitoes are the most important vectors for transmission of arthropod-borne viruses (arboviruses) that cause devastating diseases in humans and livestock (Weaver and Reisen, 2010). Both plant viruses and arboviruses can be highly pathogenic to their respective plant and vertebrate hosts. By contrast, arboviruses are known to establish persistent, non-pathogenic infections in the insect vectors to ensure maximal transmissibility (Umbach and Cullen, 2009). For plant viruses, the situation is more complex since plant viruses can be transmitted in both a non-persistent and persistent manner by insects (Dietzgen et al., 2016; explained in more detail in Section Plant Viruses). While plant viruses that replicate in insect hosts are relatively rare, they receive considerable attention in this review just because of this property: they can function as silencing vectors in insects during persistent infections. On the other hand, insect-specific viruses, with only insects as hosts, are often pathogenic although recent deep sequencing projects established the occurrence of many persistent virus infections in insects, without any apparent harmful effects on host physiology (Vasilakis and Tesh, 2015; Cory, 2016). Because of this distinction, insect-specific RNA viruses, plant viruses and arboviruses will be discussed separately.

#### Insect-specific viruses

RNA viruses that infect *Drosophila* have received much attention because of their use in the characterization of RNAi as an antiviral mechanism (reviews by Ding, 2010; van Mierlo et al., 2011; Vijayendran et al., 2013; O'Neal et al., 2014; Xu and Cherry, 2014; Gammon and Mello, 2015). The involvement of RNAi in antiviral defense was established by criteria such as (1) production of viral siRNAs (vsRNAs) by Dicer-2 during viral infection; (2) increased viral replication and mortality in RNAi mutants such as *ago-2* and *dcr-2*; and (3) production of VSR proteins as viral counterdefense. This has been mainly observed for viruses with (+)ssRNA (Flock House virus [FHV], Nodaviridae; Drosophila C virus [DCV] and Cricket Paralysis virus [CrPV], Dicistroviridae; Nora virus, unclassified, picornavirus-like) and dsRNA genome (Drosophila X virus [DXV], Birnaviridae). Interestingly, no RNAi response was reported during infections with Sigma virus, a virus with (−)ssRNA genome (Rhabdoviridae) that naturally infects *Drosophila* populations (Tsai et al., 2008; Carpenter et al., 2009), while robust RNAi signatures were observed after infections with the arbovirus Vesicular Stomatitis virus (VSV), which also belongs to the Rhabdoviridae (Mueller et al., 2010).

The induction of an RNAi response was also observed during RNA virus infection in bees (Hymenoptera; Niu et al., 2014; Brutscher et al., 2015). Interestingly, dsRNA triggers both specific and non-specific responses in bees and may therefore also act as a more general “pathogen-associated molecular pattern” (PAMP) as is observed in vertebrates (Flenniken and Andino, 2013; Piot et al., 2015; Niu et al., 2016). Honeybee colonies are typically infected by multiple viruses from the families Dicistroviridae (e.g. Israeli acute paralysis virus, IAPV) and Iflaviridae (e.g. Deformed wing virus, DWV) (Carrillo-Tripp et al., 2016). Deep sequencing of infected bees clearly shows accumulation of viral siRNAs of mainly 22 nt that are characteristic of Dicer processing (Chejanovsky et al., 2014). In another study on bumblebees, however, a clear peak of viral siRNAs was only observed during virulent IAPV infections but not in non-virulent slow bee paralysis virus (SBPV; Iflaviridae) infections (Niu et al., 2016). Furthermore, while both viral infections resulted in the increase in expression of *Dcr-2*, no effect of *Dcr-2* knockdown was observed on viral replication. These studies may point to a more minor contribution of RNAi in antiviral defense; more specifically, they may reflect the outcome of persistent viral infections which could select against a robust RNAi response (Swevers et al., 2013). Interestingly, segments of IAPV were identified in the bee genome and the expression of truncated IAPV sequences could be correlated with resistance to IAPV infection (Maori et al., 2007). While some dicistroviruses, such as DCV and CrPV, produce inhibitors of RNAi silencing (Nayak et al., 2010), it is being debated whether natural dicistrovirus infections in bees involve expression of VSR proteins (Chen Y. P. et al., 2014; Niu et al., 2016).

While hemipterans are known as vectors for transmission of plant viruses, they can also be infected by insect-specific viruses. Dicistrovirus infections were detected in the glassy-winged sharpshooter, *Homalodisca vitripennis*, and the small brown planthopper, *Laodelphax striatellus* (Nandety et al., 2013, 2015; Li J.-M. et al., 2015). Viral small RNAs were identified by deep sequencing but showed an unusual distribution: the vast majority was derived from the (+)-strand of the genome while a clear signature of Dicer cleavage (22 nt viral small RNAs) was only found for the (−)-strand. Especially in *Laodelphax* it was suggested that the viral genome was processed by not only the RNAi pathway but possibly by other degradation pathways involved (Li J.-M. et al., 2015).

In the silkworm, *Bombyx mori* (Lepidoptera), RNAi constitutes the major defense against infection of cytoplasmic polyhedrosis virus CPV (Reoviridae; Kolliopoulou et al., 2015), but as in the case of the hemipterans, alternative processing pathways of RNA/dsRNA were also detected (Zografidis et al., 2015).

Few data exist regarding the involvement of the miRNA pathway during insect-specific RNA virus infection (review by Asgari, 2015). Changes in cellular miRNAs are observed during infections with CPV (Wu et al., 2013) while the production of viral miRNAs by RNA viruses is controversial (Umbach and Cullen, 2009; Asgari, 2015).

#### Arboviruses

Arboviruses are dual-host viruses that alternate between vertebrate and insect hosts. The arboviruses comprise a wide variety of RNA virus groups such as alphaviruses (genus Alphavirus, family Togaviridae; e.g. Sindbis virus, Chikungunya virus), flaviviruses (Flaviviridae; e.g. Dengue virus, West Nile virus), bunyaviruses (Bunyaviridae; Rift Valley fever virus, La Crosse virus), rhabdoviruses (Rhabdoviridae; e.g. Vesicular Stomatitis virus) and reoviruses (Reoviridae; e.g. Bluetongue virus) (Weaver and Reisen, 2010). Arboviruses are characterized by a variety of RNA genomes: (+)ssRNA (Togaviridae, Flaviviridae), (−)ssRNA (Rhabdoviridae), segmented ambisense ssRNA (Bunyaviridae), and segmented dsRNA (Reoviridae). Because of the health risk, arbovirus infection of mosquitoes has been the focus of intense research.

Arbovirus infections generally are associated with few fitness costs that allow mosquitoes to efficiently transmit the viruses to the vertebrate host (Cheng et al., 2016). While arboviruses establish persistent infections in mosquitoes, a clear RNAi response can be detected, as evidenced by the production of viral siRNAs (reviews by O'Neal et al., 2014; Olson and Blair, 2015). Knockdown of siRNA machinery components results in more pathogenic arbovirus infections, decreased mosquito life span and decreased arbovirus transmission (Kean et al., 2015; Cheng et al., 2016). Interestingly, the piRNA pathway also contributes to the antiviral defense in mosquitoes (Bronkhorst and van Rij, 2014). Mosquitoes exhibit an expansion of Argonaute genes of the Piwi subfamily (Campbell et al., 2008). While in *Drosophila* Piwi proteins are mainly involved in defense against transposons in the germline (Brennecke et al., 2007), an antiviral role for some of the members in the expanded Piwi subfamily has evolved in mosquitoes (Miesen et al., 2015). In the C6/36 cell *Aedes albopictus* cell line, which is defective in Dcr-2 (siRNA pathway), abundant piRNAs are detected upon Dengue virus infection while siRNAs are absent (Scott et al., 2010).

The presence of VSR genes in arboviruses was first considered to be unlikely since it was thought to be incompatible with the establishment of persistent, non-pathogenic infections (Myles et al., 2008). More recently, however, mechanisms to suppress RNAi were identified during arbovirus infections, such as inhibition of Dicer activity by the NS4B protein of Dengue virus (Kakumani et al., 2013). During infection, flaviviruses also produce non-coding ssRNAs with strong secondary structures that saturate processing by Dicer (Schnettler et al., 2012). Since NSs proteins of arthropod-borne bunyaviruses are modulators of antiviral innate immune responses such as the interferon pathway in their vertebrate hosts (Eifan et al., 2013) and those of their plant counterparts have VSR activity (Hedil and Kormelink, 2016), antiviral activity may also be expected for their insect hosts (see further discussion in Section Plant viruses on plant bunyaviruses).

Very little is known about the interactions of arbovirus infections in insects with the miRNA pathway (Asgari, 2014). Changes in cellular miRNA profiles are observed after arbovirus infections of mosquitoes that can be involved in the host response to virus infection or reflect manipulation of the cellular environment by the virus. Also arbovirus-encoded miRNAs are reported although their significance remains controversial (Asgari, 2015).

#### Plant viruses

Similar to the transmission of arboviruses by mosquitoes, herbivore insects can act as vectors of plant RNA viruses. The vast majority of vectors of plant viruses consists of aphids and whiteflies (Hemiptera) with pierce-sucking mouthparts while other vector species are found among Coleoptera, Lepidoptera, Diptera, Orthoptera, Dermaptera (all with chewing mouthparts) and Thysanoptera (also with pierce-sucking mouthparts) (Fereres and Raccah, 2015). Plant viruses are reported to have more diverse modes of transmission than arboviruses (Dietzgen et al., 2016) although this may reflect differences in research focus (Blanc and Gutierrez, 2015). More specifically, plant viruses can be transmitted by insect vectors in a non-persistent, semi-persistent and non-circulative, persistent circulative and non-propagative, and persistent circulative and propagative manner (Whitfield et al., 2015; Dietzgen et al., 2016). While non-persistent and semi-persistent transmissions are carried out through external interactions with the stylet or the foregut, in persistent infections the viruses can transverse the gut barrier and invade the rest of the body before passage into the salivary glands and saliva. Circulative plant viruses that invade the insect body without viral replication belong to the families Luteoviridae [(+)ssRNA genome], Geminiviridae [single or bipartite (+)ssDNA genome] and Nanoviridae [multipartite (+)ssDNA genome] (Gray et al., 2014). Plant viruses that replicate in the insect vectors before further transmission belong to the families Reoviridae (segmented dsRNA genome), Bunyaviridae (segmented ambisense ssRNA genome) and Rhabdoviridae [(−)ssRNA genome] (Dietzgen et al., 2016). Another group of circulative propagative viruses belong to the genus *Tenuivirus* that are related to the Bunyaviridae (Whitfield et al., 2015).

While non-propagative viruses do not replicate in insect vectors, it can be assumed that no exposure of the RNA genome or replication intermediates occurs and the RNAi pathway is not activated. During propagative virus infection, however, production of viral siRNAs was reported for infections of the small brown planthopper *Laodelphax striatellus* by rice black-streaked dwarf virus (Reoviridae) and rice stripe virus (Tenuivirus) (Xu et al., 2012; Li et al., 2013), the glassy-winged sharpshooter *Homalodisca vitripennis* by *H. vitripennis* reovirus (Nandety et al., 2013), and the leafhopper *Recilia dorsalis* by rice gall dwarf virus (Reoviridae) (Lan et al., 2015). In *R. dorsalis*, knockdown of Dicer-2 resulted in decreased production of viral siRNAs concomitant with increased virus production and insect mortality (Lan et al., 2015).

While many plant viruses express VSRs during their infection of plant tissues (Pumplin and Voinnet, 2013; Zhao et al., 2016), it has not been investigated whether such VSR activity also exists in their insect vectors. The plant bunyavirus Tomato spotted wilt virus (genus *Tospovirus*) infects many species of plant, but also replicates in its thrips vectors *Frankliniella occidentalis* and *fusca*. While it has a well-described VSR in plants (Takeda et al., 2002), differences in the patterns of vsRNAs have been demonstrated between plant and thrips vector, demonstrating that vsRNA production occurs in the thrips separately from any vsRNAs ingested from the plant host (Fletcher et al., 2016). The NSs proteins of bunyaviruses have been implicated in antiviral defense in both vertebrates (as arthropod-borne virus) and plants (Hedil and Kormelink, 2016) and a similar role may be considered in insects. Similar differences in vsRNA populations have been reported for rice stripe virus (*Tenuivirus*; related to Bunyaviridae) between the plant hosts *Oryza sativa* and *Nicotiana benthamiana* and the insect host *Laodelphax striatellus* (Xu et al., 2012).

During infections with plant viruses, changes also in miRNA expression profiles were observed in planthoppers and whiteflies (Li J.-M. et al., 2015; Chang et al., 2016; Sattar and Thompson, 2016; Wang et al., 2016).

### Pathogenic and persistent infections

Viruses are well-known for causing disease (pathogenic infections) and the occurrence of persistent viral infections only recently has caught attention after the accumulation of data from deep sequencing projects. As indicated already above, arbovirus infections in mosquitoes and ticks, and some plant virus infections in hemipteran vectors can be considered as persistent infections because the viruses became adapted to the insect host physiology to ensure efficient transmission to the respective vertebrate and plant hosts. However, deep sequencing projects have revealed the presence of an enormous multitude of viral sequences in insects (Li C.-X. et al., 2015; Liu et al., 2015; Webster et al., 2015), that suggests that persistent viral infections are very common.

Such persistent infections can arise from pathogenic infections that were not cleared effectively and to which insect hosts became adapted (Goic and Saleh, 2012; Moreno-Garcia et al., 2014). Persistent infections can be beneficial to the hosts since they can provide protection against pathogenic infections by related viruses (Villarreal, 2009). Both horizontal and vertical transmissions can occur (Cory, 2016) and different viruses can interact during co-infections, resulting in shifts in infections in insect populations (Cornman et al., 2013). Deep sequencing data therefore have revealed the complexities of virus communities in natural populations of which the ecology is barely or not understood.

An interesting question is how the status of persistence is established and maintained. In both the *Drosophila* S2 cell line and mosquitoes, virus-derived DNA molecules are produced through reverse transcriptase activity of endogenous retrotransposons in the infected cells (Goic et al., 2013, 2016). The (episomic or genome-integrated) viral DNA forms subsequently produce transcripts that are processed by the RNAi machinery, resulting in control of virus replication. In several other instances, sequences of non-retro RNA viruses were found inserted into insect genomes (Crochu et al., 2004; Maori et al., 2007; Cui and Holmes, 2012; Cheng et al., 2014) and it was suggested that genomic integration could provide a defense mechanism using the RNAi mechanism, for instance through the production of piRNAs as is observed for the control of retrotransposons (Brennecke et al., 2007).

During arbovirus and plant virus infections of insects, viral siRNAs are produced as in the case of pathogenic virus infections. As a correlation exists between viral siRNA production and viral replication levels (Lan et al., 2015; Zografidis et al., 2015), it can be assumed that lower levels of siRNAs are produced during persistent infection than during pathogenic infection. Interestingly, not all viral siRNAs, which accumulate during persistent infection, appear to be functional. In S2 cells persistently infected with FHV, viral siRNAs are not associated with Ago-2-containing RNA-induced silencing complexes (RISCs) and are incapable of silencing complementary reporter constructs (Flynt et al., 2009). Silencing of RNAi components revealed also a more important role for Dcr-2 than Ago-2, indicating that Dcr-2 controls viral replication directly by its nuclease activity rather than as initiator of RNAi. Similar data were obtained in lepidopteran cell lines, where infections of FHV and Macula-like virus (derived from plant viruses of the Tymoviridae family; Katsuma et al., 2005) did not interfere with plasmid-based-expression of their capsid proteins (Swevers et al., 2016). An RNA hairpin construct against the VSR gene B2 of FHV could clear the viral infection efficiently, confirming an intact antiviral RNAi mechanism in the lepidopteran cells. Persistent FHV genomes of lepidopteran cell lines encode an intact VSR gene that can inhibit RNAi potently in a plant-based assay (Swevers et al., 2014), indicating that protection against the host RNAi machinery remains essential. Because the VSR protein B2 interacts with replication complexes of FHV in mitochondrial membranes (Aliyari et al., 2008), it likely acts locally during persistent infections and does not affect the RNAi machinery in the rest of the cytoplasm. Also a cell line from the gypsy moth, *Lymantria dispar* (Lepidoptera), was found to be persistently infected with iflavirus (Carrillo-Tripp et al., 2014). Transfection of an RNAi inhibitor in the cell line resulted in higher virus genome levels, suggesting an involvement of RNAi. Persistent infection of cell lines with RNA viruses seems to be a rather common phenomenon and therefore could function as a model to study the mechanism of RNA virus persistence (Li et al., 2007; Ma et al., 2014; Carrillo-Tripp et al., 2016; Swevers et al., 2016).

Finally, it can be noted that in some instances no activation of RNAi was observed during persistent RNA virus infections (Carpenter et al., 2009; Habayeb et al., 2009). In those cases, persistent virus infections are controlled by other innate immune pathways (e.g. Shelly et al., 2009; Nakamoto et al., 2012; Paradkar et al., 2012; Cao et al., 2016; review by Merkling and van Rij, 2013). Thus, control of virus infection is not always associated with RNAi mechanisms. Because of the general focus on the importance of the RNAi response to control viral infections, the contribution of alternative pathways may be underestimated from the literature.

### Virus specificity

Productive virus infections in insects are considered to be highly host-specific. This has been documented most extensively with baculoviruses that are used in integrated pest management as specific biological pesticides. However, the blockage of viral infection can occur at different levels of the infection process and virions may actually invade the host without generation of viral progeny (Haas-Stapleton et al., 2003; Simon et al., 2004; Wu et al., 2016). In addition, there are viruses with a wide host range, such as FHV and CrPV (Reinganum, 1975; Dasgupta et al., 2003). Illustrating further the susceptibility of insects to infection by a variety of different viruses, it has been observed that *Drosophila* can be experimentally infected by Sindbis virus, Dengue virus, West Nile virus, and Rift Valley Fever virus, that naturally infect mosquitoes and vertebrates, and recently this has emerged as a model to study arbovirus infections (Xu and Cherry, 2014). These examples make it clear that in many cases the virus-host specificity is not well-defined and that it is possible for viral particles to enter non-target hosts with unknown consequences. With the identification of many new viral species through next-generation sequencing techniques (Liu S. et al., 2011; Liu et al., 2015; Li C.-X. et al., 2015; Webster et al., 2015), it remains to be determined how specific the newly virus infections will be and how efficiently they can cross other species barriers.

## Viral genome engineering and production of recombinant virus for dsRNA delivery

If viral particles are going to be used for the delivery of dsRNA, methods should be developed for efficient introduction of gene sequences that function as template for dsRNA production. In the case of DNA viruses, this can be accomplished by the introduction of inverted or everted repeat sequences downstream of a strong viral promoter such that RNA hairpins with dsRNA structure are produced during viral infection (Kennerdell and Carthew, 2000). For RNA viruses with ssRNA genomes, single gene sequences integrated in the viral genome will become double-stranded during genome replication (Gammon and Mello, 2015), while for dsRNA viruses both genomes and replication intermediates can function as triggers of RNAi (Zografidis et al., 2015). RNA viruses with ssRNA genome can also produce (single-stranded) structured RNAs that are substrates of Dicer enzymes (Schnettler et al., 2012).

### Baculoviruses

During the last decades, the baculovirus expression system, based on AcMNPV, has evolved into a major platform for production of recombinant proteins (van Oers et al., 2015). Important applications of the baculovirus expression system include the production of vaccines and gene therapy vectors based on adeno-associated virus (Felberbaum, 2015). The availability of commercial platforms for production of recombinant AcMNPV also allows the rapid generation of baculoviruses expressing RNA hairpins. In insect culture cells, baculoviruses expressing dsGFP were reported to silence GFP fluorescence (Huang et al., 2007). Silencing effects were also observed with antisense constructs (Hajos et al., 1999). However, baculoviruses generally are pathogenic and effects of gene silencing can be masked by non-specific toxic effects. When recombinant BmNPV (also generated by bacmid technology; Motohashi et al., 2005) was used to deliver dsRNA targeting a juvenile hormone esterase-related (JHER) gene in the corn borer, *Sesamia nonagrioides*, many non-specific phenotypic effects associated with disrupted metamorphosis were observed (as also observed in BmNPV expressing control dsRNA; Kontogiannatos et al., 2013), despite the restricted host specificity of BmNPV (Maeda et al., 1993). Only through the analysis of a large number of animals could specific phenotypes be identified that were associated with knockdown of JHER (Kontogiannatos et al., 2013). A solution for this drawback could be the generation of incapacitated baculoviruses, i.e., baculoviruses that are deficient for an essential gene in the infection cycle, such as *ie-1* or *lef-8* (Efrose et al., 2010; Ioannidis et al., 2016). Incapacitated baculoviruses are predicted to enter target cells and initiate early gene expression without progressing to the late phase of the infection cycle and cell lysis. For gene transduction purposes, incapacitated baculoviruses should therefore be engineered with expression cassettes with constitutive cellular or early viral promoters instead of the very late promoters used for protein expression. However, the production of virions that contain deficient genomes in rescue cell lines, which provide the missing functions as transgenes, has turned out to be challenging and only low titers of incapacitated baculoviruses could be produced (Efrose et al., 2010; Ioannidis et al., 2016). The use of baculoviruses for transduction of dsRNA in insects therefore requires the isolation of variants/mutants with low virulence that can be easily manipulated genetically and produced in cell lines.

### Densoviruses

Plasmids that carry densovirus genomes have been used to produce infectious particles after injection into susceptible insect hosts or after transfection of cell lines (Afanasiev et al., 1999; Ren et al., 2008; Suzuki et al., 2014). Interestingly, plasmids containing densovirus sequences can integrate into the cellular genome and copy numbers increase upon deletion of the non-structural genes (Bossin et al., 2003).

**Figure 1:**
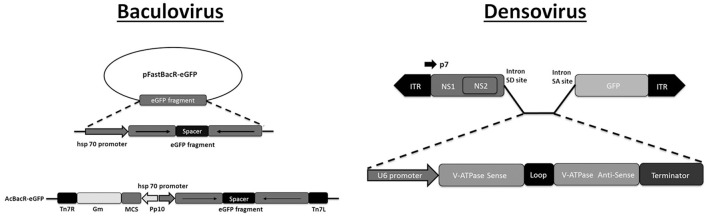
Plasmid-based systems for production of recombinant DNA virus. **Baculovirus**. The pFastBac vector is modified with an expression cassette of an RNA hairpin (targeting GFP) under the control of the hsp70 promoter. The cassette is subsequently transferred from the pFastBac vector to the baculovirus genome using bacmid technology (Bac-to-Bac system, Invitrogen). Adapted with permission from Huang et al. (2007). Abbreviations: Gm, gentamycin resistance cassette; Tn7L and Tn7R, Tn7 transposase recognition sites. **Densovirus**. The shRNA expression cassette is inserted into an artificial intron that is introduced to an engineered densovirus genome. The shRNA cassette is under control of an RNA polymerase III promoter (U6) and targets the V-ATPase gene. Infectious virions are produced after co-transfection with a plasmid of the engineered genome and a plasmid that contains the wild-type genome that expresses the capsid protein (not shown). Adapted with permission from Gu et al. (2011). Abbreviations: ITR, inverted terminal repeat; NS1 and NS2, non-structural genes 1 and 2; SD and SA, splice donor and acceptor. P7 refers to the promoter for the non-structural genes.

Recombinant densovirus vectors (Brevidensovirus genus) were constructed for delivery of short RNA hairpins in mosquitoes (Gu et al., 2011). *Aedes albopictus* C6/36 cells were used for production of recombinant virions which required co-transfection with plasmids containing intact densovirus genomes (resulting in production of helper viruses). In recombinant virus genomes, an intron was inserted between the ORF of the non-structural gene 1 (NS1) and a GFP cassette, that contained a RNA polymerase III-driven short hairpin RNA (shRNA) cassette (Figure 1). Transcription by the viral promoter results in the production of a pre-mRNA which is spliced to generate mRNA for functional NS1-GFP proteins (and green fluorescence) while the intronic expression cassette functions as a source of siRNA. When *Ae. albopictus* mosquito larvae were exposed to mixtures of helper and recombinant densovirus with the shRNA cassette targeting *V-ATPase*, silencing of the targeted gene was observed together with increased mortality. Monitoring of GFP fluorescence after infection demonstrated that densovirus could infect many different mosquito tissues (Gu et al., 2011).

Densoviruses as a group have a wide distribution among insect species and were reported to occur in representatives of many insect orders (Liu K. et al., 2011; Han et al., 2013). However, mosquito densoviruses are highly specific to mosquitoes (Suzuki et al., 2015). Specificity and virulence toward different hosts seem to be determined by residues in the capsid sequences (Multeau et al., 2012). Despite their sometimes high virulence in insects, densoviruses are not known to infect mammals (Carlson et al., 2006; Jiang et al., 2007). On the other hand, densoviruses can also occur as persistent infections (and being vertically transmitted) and can make insect hosts refractory to other pathogens (Xu et al., 2014). In summary, densoviruses represent a group of insect DNA viruses which have a limited host range, are capable of different infection states and can be easily manipulated and produced in cell lines. These properties will drive the research on densoviruses for applications in environmentally safe pest control, and as gene transduction and RNAi delivery vectors.

### Other DNA viruses

Although tedious, production of recombinant DNA virus is straightforward if an *in vitro* culture system is available. This is illustrated by the generation of recombinant iridovirus in a coleopteran cell line (Ozgen et al., 2014). After identification of a locus in the viral genome of which the inactivation would not interfere with the infection cycle, a transfer vector is created consisting of a plasmid that contains the homologous viral regions flanking the insertion site of the transgene. For applications as RNAi delivery vector, the transgene consists of an RNA hairpin expression cassette, perhaps in combination with a fluorescence reporter cassette to facilitate selection of recombinant virus. After transfection of the transfer vector in virus-infected cells, fluorescent viruses are generated by homologous recombination between the transfer vector and the wild-type DNA virus. Recombinant viruses are subsequently purified by plaque purification (O'Reilly et al., 1992).

### Insect-specific RNA viruses and arboviruses

As is the case for DNA viruses, production of recombinant RNA viruses typically requires the cloning of the genome which then can be manipulated and expressed using a suitable promoter. However, efficient amplification and packaging of linear viral RNA genomes in capsids, and eventually envelopes, to produce infectious virions typically requires precise 5′- and 3′-ends in the viral genomic RNAs. In the case of FHV (Nodaviridae), 5′-extensions are minimized by placing the promoter such that transcripts are initiated at or very close to the authentic 5′-end of the viral genome, while precise 3′-ends are generated by cleavage of appropriately positioned hepatitis delta virus ribozyme (Ball and Johnson, 1999; van Cleef et al., 2014). In addition, the ends of linear RNA genomes often form strong secondary structures which complicate the isolation of 5′- and 3′-ends (Carrillo-Tripp et al., 2015). Picorna-like viruses, such as iflaviruses and dicistroviruses, often occur as quasi-species in which multiple viral forms complement each other to support infection that prevents the isolation of efficient individual infectious sequences (Ojosnegros et al., 2011) (see also further below). Such features make the construction of infectious cDNAs of RNA viruses challenging, even if it concerns small genome sizes. However, a population cloning strategy in which multiple independent clones of the 5′ part of the genome are ligated to multiple clones representing the 3′ part may make it possible to recover infectious clones with different properties. For the plant potexvirus Alternanthera mosaic virus (AltMV), this strategy allowed the isolation of multiple virus clones that differ in replication and silencing suppression (Lim et al., 2010b). Different RNA silencing suppression efficacy in the clones could be correlated with their suitability for virus-induced silencing (VIGS) or protein expression applications (Lim et al., 2010a).

Another obstacle that prevents the establishment of plasmid-based production systems of RNA viruses is the availability of suitable insect cell lines. The vast majority of cell lines is derived from lepidopteran and dipteran insects (Lynn, 2001), while more recently cell lines have been reported from coleopterans and hymenopterans (Smagghe et al., 2009; Goodman et al., 2012; Goblirsch et al., 2013). Cell lines derived from insects of other orders are very rare (Boyapalle et al., 2008). Viruses may be restricted to particular cellular environments (from single or related host species) for robust replication and virion reproduction. In case of adaption to more widely used cell lines, danger exists for decrease in infectivity of the original insect host. It is also observed that species-specific viruses have great difficulties to replicate in cell lines derived from the host. For instance, an *in vitro* cell culture system for CPV (Reoviridae) in insects has not been reported despite the availability of many host cell lines (Hill et al., 1999; Swevers et al., 2014). This contrasts with the existence of reverse genetic systems for reoviruses using mammalian cell lines (Boyce et al., 2008). For viruses that are difficult to grow in cell lines, tissue culture has been used as an alternative (Arif and Pavlik, 2013).

Because of its importance with respect to human and animal health, considerable effort has been paid to the development of infectious clones and reverse genetics systems for arboviruses that cause disease in humans and livestock (Aubry et al., 2015). Such systems were developed some time ago based on mammalian cell lines for alphaviruses (Sindbis virus; Bredenbeek et al., 1993), flaviviruses (Yellow Fever virus, Dengue virus; Ruggli and Rice, 1999), rhabdoviruses (Vesicular Stomatitis Virus; Lawson et al., 1995), bunyaviruses (model Bunyamwera virus; Bridgen and Elliott, 1996), and also more recently reoviruses (Blue Tongue virus; Boyce et al., 2008). Recombinant arboviruses generated from infectious clones were also used to study infection in mosquito cell lines and mosquitoes (e.g. Siu et al., 2011; Slonchak et al., 2014). In particular, a Sindbis virus expression system was developed, in which engineered Sindbis viruses contain a second subgenomic RNA promoter element that can be used for protein or antisense RNA expression (Johnson et al., 1999; Foy et al., 2004). Engineered Sindbis viruses were used to deliver RNAi in mosquitoes and the silkworm *Bombyx mori* (Adelman et al., 2001; Shiao et al., 2001; Uhlirova et al., 2003). Because of potential health hazard, however, manipulation of arboviral genomes and production of infectious virions require containment procedures of biosafety level 2 or higher. Safety issues will also prevent the use of arbovirus vectors as RNAi delivery vehicles in insect (mosquito) populations in nature.

**Figure 2:**
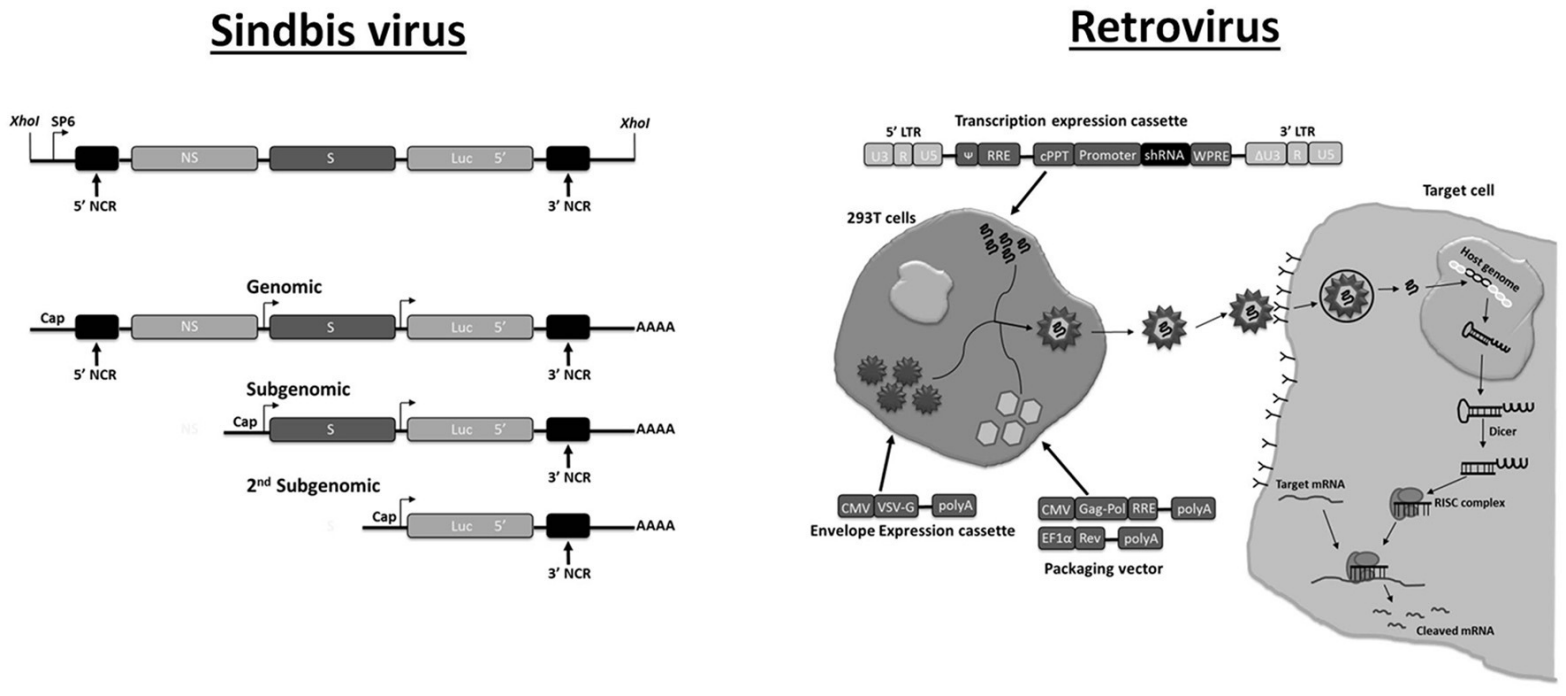
Plasmid-based systems for production of recombinant RNA virus in mammalian cells. **Sindbis virus**. The DNA segment is shown that produces infectious recombinant RNA virus genome after Sp6 RNA polymerase transcription *in vitro* (top). The recombinant RNA genome contains an additional expression cassette for antisense *Luc* RNA under the control of a second subgenomic promoter. During viral replication, replication intermediates with dsRNA structures are produced that will trigger silencing of the luciferase reporter. The different transcripts (genomic, subgenomic, second subgenomic) that are produced during infection are shown at the bottom. Adapted with permission from Johnson et al. (1999). Abbreviations: NCR, non-coding region; NS, non-structural; S, structural. **Retrovirus**. A non-replicating lentiviral vector is shown that contains a shRNA expression construct (“transcription expression cassette”). To produce virions, suitable (mammalian) cell lines are co-transfected with two other plasmids containing a packaging cassette (“packaging vector”) and an envelope expression cassette. The generated lentiviral virion is used to transduce target cells for shRNA expression. The vector containing the shRNA expression cassette is integrated in the host cell genome to express shRNA but infectious virus is not produced because of the absence of structural genes. The system can be modified to target insect cells by generating virions that produce an envelope protein which can infect almost all cell types (such as vesicular stomatitis virus glycoprotein; VSV-G). Adapted with permission from Manjunath et al. (2009). Abbreviations: LTR, long terminal repeat; ψ, packaging signal; cPPT, central polypurine tract; RRε, Rev response element; WPRE, woodchuck hepatitis virus post-transcriptional regulatory element; CMV, cytomegalovirus promoter; EF-1α, elongation factor-1α promoter; Gag-Pol and Rev refer to lentiviral genes that are essential for producing functional virions (provided in trans to the “transgene expression cassette”).

Similar safety issues apply for the use of pseudo-typed mammalian retroviruses that were used in the past as gene transduction vectors in *Drosophila* and mosquitoes (Matsubara et al., 1996; Jordan et al., 1998; Teysset et al., 1998). After infection, retroviral genomes undergo reverse transcription into a DNA form which subsequently integrates in the cellular genome as a provirus. Proviruses are transcribed as cellular genes, and retroviral vectors engineered with RNA hairpin cassettes therefore could be applied as a permanent means of dsRNA production in infected cells. Interest for pseudo-typed “pantropic” retroviruses declined because of difficulties in production of virions at high titers. More recently, there exists renewed interest for pseudo-typed mammalian retroviruses as a transgenesis system in worms (Mann et al., 2014; Hagen et al., 2015). Retrovirus-based vectors have been used extensively in mammalian systems and were applied to perform large scale screens to analyze basic physiological processes and investigate disease mechanisms (Root et al., 2006; Manjunath et al., 2009) (Figure 2).

**Figure 3:**
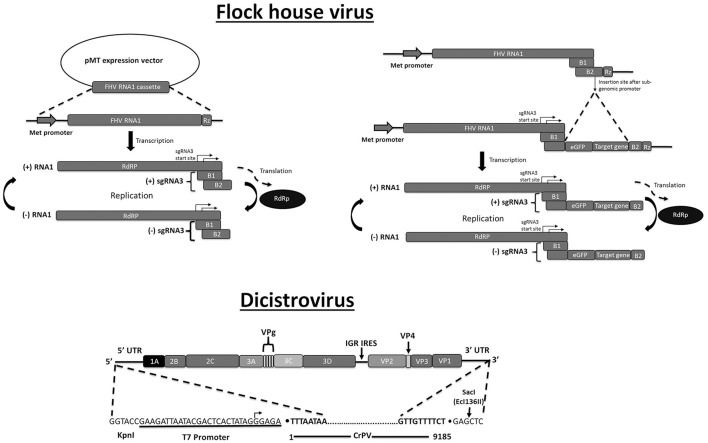
Plasmid-based systems for production of recombinant RNA virus in insect cells. Flock house virus. **Left:** FHV RNA1 can be produced in Drosophila S2 cells using the pMT plasmid that contains the sequences of the FHV RNA1 genomic fragment between the inducible metallothionein (Met) promoter and the ribozyme (Rz) cleavage site. RNA-dependent RNA polymerase (RdRp), encoded by the long ORF, will drive replication of genomic RNA 1 and subgenomic RNA3 (sgRNA3). SgRNA3 encodes two proteins, B1 (unknown function) and B2 (RNAi inhibitor). **Right:** Recombinant sequences can be inserted in the B2 ORF (downstream of the RdRp ORF) such as eGFP (in-frame with B2) and a RNA hairpin (“target gene”). For production of virions, an expression construct for RNA2, encoding the capsid protein, needs to be provided (not shown). **Dicistrovirus:** Full-length Cricket paralysis virus (CrPV) is produced by *in vitro* transcription using T7 RNA polymerase. Indicated are the transcription start site and the Ecl136II restriction site that is used for linearization of the plasmid (defining the 3′-end). The two ORFs of the dicistrovirus genome are indicated encoding the non-structural proteins (1A, 2B, 2C, 3A, 3C, 3D) and the structural proteins (VP1, VP2, VP3, VP4). VPg refers to the 5′ viral protein cap. Adapted with permission from Kerr et al. (2015). Abbreviation: IGR IRES, intergenic region internal ribosome-entry site.

Plasmid-based systems were developed for FHV (Nodaviridae) that permit robust replication in a variety of organisms including insects, yeast and plants (Selling et al., 1990; Price et al., 1996; van Cleef et al., 2014). The (+)ssRNA genome of FHV consists of two segments that encode the RNA-dependent polymerase and the capsid protein, respectively. During replication, subgenomic RNA3 is generated that produces the B2 RNAi inhibitor (Venter and Schneemann, 2008). An expression cassette that transcribes RNA1 with precise 5′- and 3′-ends, realized by positioning of the promoter sequence and self-cleaving ribozyme, respectively, can initiate high levels of FHV replication (Figure 3). In the presence of RNA2, the replication system will generate functional virions. The FHV replication/production system has been modified to drive GFP expression in infected cells, either through insertion in the 3′-part of RNA1 (Price et al., 2000) or through modified (defective) RNA2 molecules (Dasgupta et al., 2003). FHV infection results in production of viral siRNAs (Gammon and Mello, 2015) and insertion of foreign sequences in the FHV genome could therefore be employed to deliver specific RNAi effects in infected cells.

Two other important (+)ssRNA virus families that are known for their ubiquitous infections of insects are dicistroviruses and iflaviruses (Picornavirales order) (Ryabov, 2016). Both types of virus have a (+)ssRNA genome of 9–10 kb containing an attached viral protein (VPg) at the 5′-end and a poly(A)-tail at the 3′-end. Viral proteins are expressed after proteolytical cleavage of polyproteins encoded by one (iflaviruses) or two (dicistroviruses) long ORFs. Their occurrence in bees has been well studied because of possible links with colony-collapse disorder in the honeybee and declining health of pollinators (Niu et al., 2014; McMenamin and Genersch, 2015; Gisder and Genersch, 2016). Full-length clones were reported for the genomes of dicistroviruses infecting the Bird cherry-oat aphid *Rhopalosiphum padi* (RhPV) and the honeybee (Black queen-cell virus, BQCV), but their capacity to produce infectious virions was not established conclusively (Benjeddou et al., 2002; Boyapalle et al., 2008; Pal et al., 2014; Carrillo-Tripp et al., 2015). Only very recently infectious genomic RNA was produced by T7 RNA polymerase transcription of cloned sequences of the model dicistrovirus CrPV (Kerr et al., 2015). As is the case for FHV, insertion sites for foreign sequences that do not disrupt the viral function need to be identified for the generation of recombinant reporter viruses or RNAi delivery vectors.

An important question is to what extent siRNAs are produced after infection of insect-specific RNA viruses. RNA viruses encode VSRs that prevent the activation of the RNAi mechanism (Ding and Voinnet, 2007; Vijayendran et al., 2013; Gammon and Mello, 2015; Olson and Blair, 2015). As for baculoviruses, general pathogenic effects may mask the RNAi response. During persistent infections, on the other hand, Dicer-produced siRNAs may not become loaded in RISC complexes, limiting the extent of silencing the target genes (Flynt et al., 2009; Swevers et al., 2016). SiRNAs may not be produced evenly across the length of the viral genomes (e.g. for FHV Aliyari et al., 2008; Flynt et al., 2009) and insertion in particular “hotspot” regions may trigger a more effective silencing mechanism.

Studies of the effect of VSRs on VIGS during virus infection in insects are rare. One example is the incorporation of the B2 RNAi suppressor of FHV in the genome of Sindbis virus, an arbovirus without known RNAi suppression activity, which resulted in high mortality and suppression of accumulation of vsRNAs (Myles et al., 2008). Differences in pathogenicity between the dicistroviruses DCV and CrPV were also attributed to the strength of the encoded VSRs (Nayak et al., 2010). The balance between VSR potency and efficiency of viral replication kinetics is therefore considered as a major determinant for the outcome (persistent or pathogenic) of a viral infection (O'Neal et al., 2014). On the other hand, more data are available on the effect of VSR on VIGS in plants. For example, synergism between Tobacco etch virus (TEV) and Potato X virus (PXV) is caused by the efficient VSR activity of the TEV-encoded helper component proteinase (HC-Pro) which acts to protect and enhance replication of co-infecting PVX (Shi et al., 1997). Mutations in HC-Pro that are correlated with decreased VSR activity are also responsible for decreases in viral pathogenicity in other potyviruses (Shiboleth et al., 2007; Seo et al., 2011; Li M.-J. et al., 2014). As already mentioned above, variability in efficacy of the VSR affects the applicability of AltMV for protein expression vs. VIGS in plant tissue (Lim et al., 2010a,b). While parallels may exist between insect and plant infections, differences in the RNAi machinery between the two need to be considered, such as the existence of multiple dicer enzymes and the amplification mechanism by RNA-dependent RNA polymerase in plants (Siomi and Siomi, 2009), which likely make the RNAi response more powerful and therefore more subjected to control mechanisms.

Finally, more sophisticated approaches may be needed that go beyond simply inserting a target sequence in the viral genomes. One example is the insertion of a functional miRNA-expressing cassette in an intron of one of the segments of influenza viral genome which was used to silence a GFP reporter in mammalian cells (Varble et al., 2010). Influenza virus (Orthomyxoviridae) however replicates in the nucleus and therefore will provide a more natural environment for processing miRNA-like genes when compared to RNA viruses that replicate in the cytoplasm (Asgari, 2015). All these considerations underline the immaturity of the field and major research efforts are required to investigate the suitability of the approach which may depend on the type of virus and the type of infection.

### Plant viruses

**Figure 4:**
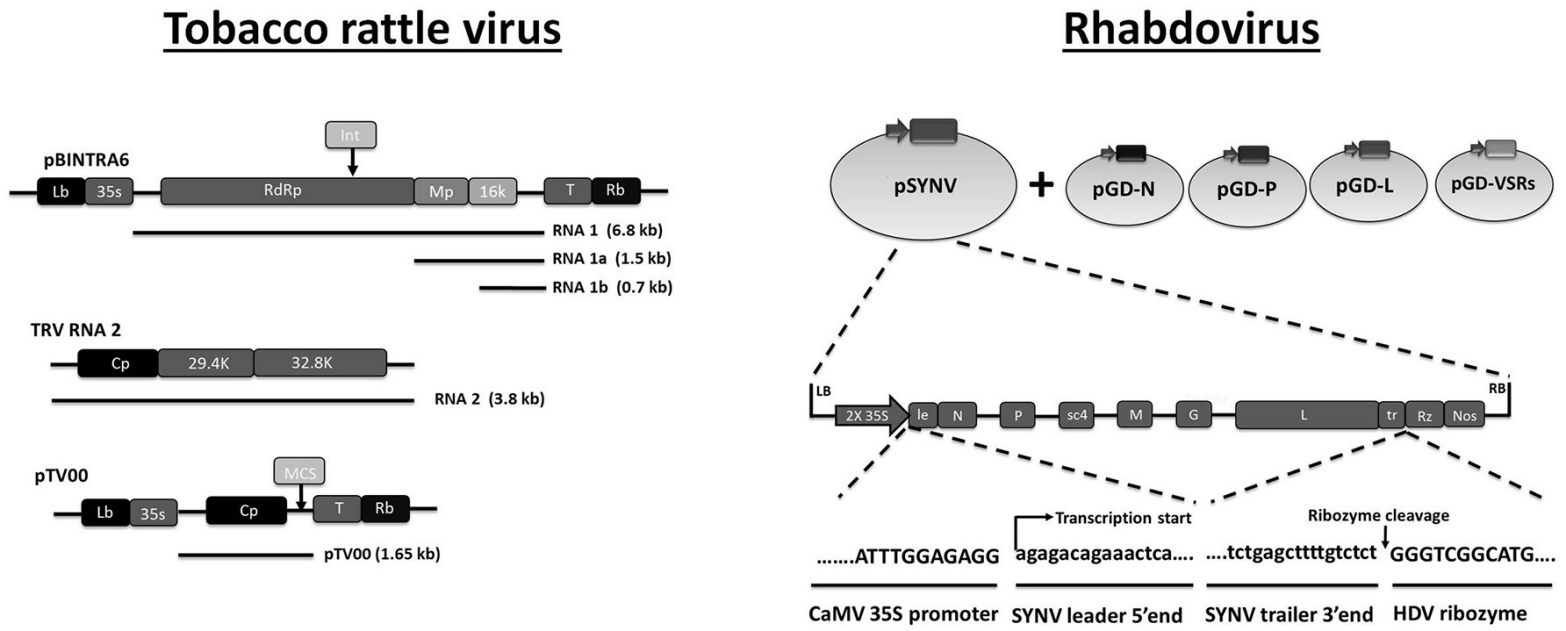
Plasmid-based systems for production of recombinant RNA virus in plant cells. It is noted that the two plant virus systems presented here are expected to deliver dsRNA according to different mechanisms. Tobacco rattle virus (TRV) is used as a VDPS in plant cells, which means that large amounts of dsRNA are produced within the plant cells that subsequently are taken up by the insect during feeding. TRV itself does not replicate in insects. Sonchus yellow net rhabdovirus (SYNV), on the other hand, is expected to replicate in both plant and insect cells. As such it represents a true VIGS system for insects since dsRNA production occurs during replication in insect tissues. **Tobacco rattle virus:** At the top is shown the T-DNA organization of pBINTRA6, a plant binary transformation vector containing TRV RNA1. pTV100 (bottom) is a binary transformation vector that contains an engineered sequence of TRV RNA2. More specifically, the non-essential 29.4 and 32.8 k genes of wild-type TRV RNA2 (middle) were replaced with a multiple cloning site (MCS) that can be used for insertion of reporter genes and sequences for targeting foreign genes through RNAi. To generate virus-induced gene silencing (VIGS) or viral dsRNA production to target insect pests, leaves of *Nicotiana benthamiana* are infiltrated with a mixture of *Agrobacterium* containing pBINTRA6 and pTV100. Adapted with permission from Ratcliff et al. (2001). Abbreviations: Lb and Rb, left border and right border of the T DNA; 35S, Cauliflower mosaic virus 35S promoter; RdRp, RNA-dependent RNA polymerase; Mp, movement protein; 16 k, 29.4, 32.8 refer to proteins of the indicated MW; Cp, capsid; T, terminator; Int refers to the position of an intron that is introduced for stability of the construct in *E. coli*. **Rhabdovirus:** Generation of an infectious clone of Sonchus yellow net rhabdovirus (SYNV). Plasmid pSYNV contains the full antigenome of SYNV between the double 35S (2x35S) promoter and the hepatitis delta virus (HDV) ribosome (Rz) in the plant binary transformation vector. The supporting plasmids pGD-N, pGD-P and pGD-L encode the nucleoprotein (N), phosphoprotein (P), and large RNA polymerase (L) core proteins needed for nucleocapsid formation while the pGD-VSR plasmids encode viral suppressors of RNAi silencing. Adapted with permission from Wang et al. (2015). Abbreviations: Lb and Rb, left border and right border of the T DNA; le, leader; tr, trailer; Nos, nopaline synthase terminator; sc4, movement protein; G, glycoprotein; M, matrix protein.

Plant viruses are discussed separately because robust production systems are typically available using plants. Agro-infiltration is being used for high efficiency delivery of recombinant plant virus genomes in plant tissue (Mallory et al., 2002; Marillonnet et al., 2005; Lindbo, 2007). Besides for protein production (Gleba et al., 2007), viral expression systems, e.g. based on tobacco mosaic virus (TMV) and potato virus X (PVX), were also used to study phenotypic effects of specific gene silencing in plants (virus-induced gene silencing, VIGS; Kumagai et al., 1995; Ruiz et al., 1998; Purkayastha and Dasgupta, 2009; Lange et al., 2013). Because the recombinant viruses carry an insert corresponding to a cellular gene, the RNAi response will silence both viral genes and the host gene targeted by the insert. A more recent application is the use of plant viral expression vectors as viral dsRNA production systems (VDPS; Kumar et al., 2012). In this case, additional dsRNA molecules produced during viral replication are not targeted to endogenous plant genes but to genes of herbivore nematodes and insects to provide protection against feeding (Dubreuil et al., 2009; Kumar et al., 2012). Vectors based on tobacco rattle virus (Figure 4) are preferred because mild disease symptoms are induced and expression is both more intense and more persistent (Ratcliff et al., 2001). However, different plant virus expression systems may need to be tested for different host plant-insect pest systems to achieve optimal performance (Wuriyanghan and Falk, 2013). For instance, viruses that replicate preferentially in the phloem, such as the citrus tristeza virus (CTV), were engineered in similar fashion to provide protection against the Asian citrus psyllid *Diaphorina citri*, which feeds on the phloem (Hajeri et al., 2014). Several more examples of the application of plant viruses to affect mortality or fecundity of insect pests include the use of recombinant TMV in the caterpillar *Mythimna separata* and the citrus mealybug *Planococcus citri* (Khan et al., 2013; Bao et al., 2016) and of PVX in the solenopsis mealybug, *Phenacoccus solenopsis* (Khan et al., 2015). A high-throughput Gateway-enhanced AltMV system was also described to test RNAi constructs from a cDNA library for effects on growth and fecundity in the whitefly, *Bemisia tabaci* (Ko et al., 2015). Because the transgenic approach of plant-mediated RNAi requires a long term of development, the use of plant viral vectors provides a fast-track approach for testing the efficiency of silencing of candidate genes and induction of associated toxic effects.

However, the VDPS systems mentioned above cannot be viewed as strict viral delivery systems of RNAi. The plant viruses are rather used as tools to produce dsRNA in plant cells at sufficient levels to cause silencing effects in the insect after feeding on the plant tissue. Viral delivery is here viewed as the use of viruses to transmit the RNAi signal, which is typically realized after infection of insect tissues by viral particles and when viral replication occurs in insect cells. Since most plant viruses do not replicate in insect tissues, they cannot be regarded as strict viral delivery systems but merely as dsRNA production systems in plant tissue. Only plant viruses that can penetrate the gut barrier are therefore considered as engaged in “viral delivery of dsRNA.”

As discussed already above, persistent circulative plant viruses are capable to transverse the gut-hemocoel barrier. Of these, non-propagative viruses transverse different tissues without replication and it is not clear whether active dismantling of virions occurs during passage. This more passive behavior resembles that of “virus-like particles” which will be discussed in the next section. A few reports however indicated the occurrence of replication in insect vectors of viruses commonly considered to be transmitted in a non-propagative manner (Rivera and Gamez, 1986; Pakkianathan et al., 2015). During passage through the insect body, it can be expected that cells become infected, virions are dismantled and moderate viral replication may be initiated. Such considerations invite research that (re-)addresses the capacity of plant viruses to replicate in insects and can lead to surprising findings, such as the recent report of replication and virion production of tobacco ringspot virus (Picornavirales) in honeybees and their *Varroa* mite parasites (Li J. L. et al., 2014); but which is contested (Miller et al., 2014). As previously mentioned, *B. mori*-derived BmN cells and other lepidopteran cell lines can be persistently infected by Macula-like virus that is related to plant Tymoviruses and apparently has crossed the plant-insect species barrier (Katsuma et al., 2005). However, virus replication may not at all be necessary for “survival” of the virus during the passage of the insect body. It has also been reported some time ago that specific interactions between the luteovirus potato leafroll virus and GroEL, a chaperone-like protein produced in the aphid haemolymph by endosymbiotic Buchnera bacteria, protect virus particles from degradation in the hemocoel of the aphid when it is transported to the salivary glands of the aphid (Hogenhout et al., 1998).

Plant viruses that are transmitted by insect vectors in a persistent circulative and propagative manner are the most interesting candidates since active viral replication, and consequently amplification of the RNAi response, will occur in the insect hosts. Circulative and propagative viruses belong to the families Bunyaviridae, Reoviridae, and Rhabdoviridae, and have a complex (−)ssRNA, segmented ambisense RNA or segmented dsRNA genome structure which has made the generation of infectious clones and the development of reverse genetics systems challenging. Only very recently a reverse genetics system was developed for Sonchus yellow net rhabdovirus (SYNV; Wang et al., 2015). The procedure involves agro-infiltration of tobacco plants of a construct expressing the negative strand of the viral genome together with co-expression of nucleoprotein, phosphoprotein, core polymerase and suppressors of RNAi (Figure 4). Recombinant SYNV expressing GFP was generated and used to investigate the functions of the movement protein and the glycoprotein (Wang et al., 2015). It will be interesting to see how the reporter SYNV infects the insect vector and whether delivery of RNAi triggers is possible. A shortcoming for this research also is that the production of rhabdoviral virions occurs in a plant system. There is a need for the development of insect cell lines that are capable to support replication and virion production of plant viruses to study their infection cycle in the insect vectors (Ma et al., 2013). This is feasible since bunyaviruses, reoviruses, and rhabdoviruses occur also as insect-specific viruses and arboviruses, and therefore likely originated in their insect hosts.

Interestingly, plants are capable of producing insect viruses with a broad host range, such as FHV (Nodaviridae). High titers of FHV could be generated in transgenic tobacco plants that express movement proteins of plant viruses (Dasgupta et al., 2001). It is speculated that FHV originated from recombination events between a plant virus and a virus from a plant-feeding insect, which would explain its wide host range. Although it replicates in insects (Dasgupta et al., 2007), FHV is not known to be transmitted by them, while infection by mechanical disruption is possible.

## Virus-like particles

Virus-like particles (VLPs) are molecular vehicles assembled from key structural components of viral origin that have been repurposed to deliver a cargo different from the initial viral genome. The VLPs' components are proteins which participate in the formation of the viral capsid, and sometimes of the envelope as well. Although they do not retain their infectious properties, VLPs are empty shells that are able to enter the respective target cells (Ludwig and Wagner, 2007; Lund et al., 2010). There are several systems of different origins that have been proposed as adequate platforms for the production of VLPs, such as prokaryotes, eukaryotes, viruses, as well as cell-free and *in vitro* assembly conditions (Shirbaghaee and Bolhassani, 2016).

Agricultural biotechnology could use VLPs and exploit certain properties that these particles possess, such as their capacity for packaging of foreign RNA. A popular system applied toward this direction is the TMV particle, which consists of its coat protein (CP) and has been the first macromolecular structure identified as capable of self-assembling *in vitro*. TMV carries a 300 nt stem-loop signal sequence in its RNA (origin of assembly, Oa), that interacts with CP to initiate packaging and capsid formation. Importantly, propagation of packaging in TMV is independent of RNA sequence, allowing for packaging of heterologous RNAs since only the initial nucleation requires the Oa sequence, while the process of elongation of packaging is independent of the sequence (Butler, 1999).

When genomes of the arbovirus Semliki Forest Virus (SFV) were engineered to contain the Oa sequence of TMV, they could be trans-packaged *in vitro* inside the capsid protein of TMV forming pseudo-virions. The pseudo-virions could infect mammalian cells to express a reporter gene and were also used successfully for immunization (Smith et al., 2007). Similarly, another study employed instead FHV, whose packaging normally depends on its autonomous replication, being directed by a self-encoded RNA-dependent RNA polymerase, and its specificity is orchestrated by the synchronized transcription and translation of CP mRNA (Annamalai et al., 2008). Interestingly, it was demonstrated that replication-independent genome packaging is possible upon capsid self-assembly in a study where TMV pseudo-virions could efficiently trans-encapsidate the genome of FHV virus engineered to express green fluorescent protein (GFP) and the Oa signal sequence of TMV (Maharaj et al., 2014). Despite efficiency in encapsidation, TMV does not infect or transverse the gut in insects and further engineering is required to achieve this goal, for instance by incorporation of peptide sequences in the CP protein to allow interaction with receptors in the insect gut epithelial cells.

Another system widely used is the bacterial production of VLPs, with recent progress achieved especially in the RNAi field (APSE technology; www.apsellc.com). This system involves the production of APSE RNA Containers (ARCs) by *E. coli* bacteria which are transformed with a plasmid encoding naturally occurring proteins, such as CPs from bacteriophage MS2. Regarding the RNAi signal, the same bacteria are co-transformed with a plasmid that codes for an RNA precursor sequence (i.e., siRNA, shRNA, miRNA) together with a packaging site. After culture, the double-transformed *E. coli* are purified, leading to a large scale production of self-assembled nanocontainers which have encapsidated the desired small RNA molecule. The ARCs are expected to be rapidly taken up by the insect when sprayed; being also resistant to hydrolases, the ARCs offer environmental stability.

Viral CPs can be produced and purified using different expression systems such as bacteria, plants and insect cells (Ren et al., 2006; Moon et al., 2014; Hassani-Mehraban et al., 2015; Mendes et al., 2015). They can subsequently be assembled efficiently *in vitro* to VLPs and encapsidate under specific conditions cargo materials such as ssRNA (Aniagyei et al., 2008). Cowpea chlorotic mottle virus (CCMV; Bromoviridae) VLPs, for instance, have been shown to incorporate heterologous ssRNAs of different sizes (Cadena-Nava et al., 2012). Plant VLPs have also been successfully engineered as scaffold for epitope presentations (Hassani-Mehraban et al., 2015 and references therein). While mainly intended to be used for immunization and vaccine production in mammals, future epitope insertions could target specific receptors in the insect gut to stimulate uptake [for instance, using phage-assisted continuous evolution (PACE); Dovrat and Aharoni, 2016].

Also the baculovirus expression system has been used for production of VLPs based on insect viruses [e.g. nodavirus (Krishna et al., 2003); bidensovirus (Pan et al., 2014); dicistrovirus (Ren et al., 2014); tetravirus (Mendes et al., 2015); and alphavirus (Hikke et al., 2016)] and these could be directly used as specific delivery vehicles when loading with specific cargo is achieved. After assembly of the VLPs and their cargo *in vitro*, they could be further purified and directly applied to insects to investigate insecticidal effects.

Considering the value of protection of humans and livestock animals in combination with the cost of producing and purifying VLPs, it is likely that VLPs will be more valuable in control of insects attacking mammalian hosts (e.g. mosquitoes). On the other hand, for treatment of crop plants, replicating plant viruses in plant tissue will have more advantages than VLPs, including the relatively low cost of increasing production of the viral inoculum (and lack of necessity to obtain highly purified preparations for inoculation) and the absence of requirement for environmental stabilization. By contrast, production of the quantities of VLPs necessary to spray at a field scale in agriculture for insect control would not be a trivial challenge and needs to be set up under controlled greenhouse conditions.

Main challenges in the field are, first, whether dsRNAs or hairpin RNAs, with strong structural properties, can be successfully incorporated in capsids of viruses with ssRNA genomes (which are mostly used as the basis for production of VLPs), and second, whether assembly of VLPs with dsRNA cargo can directly be achieved in appropriately engineered transgenic plants (as opposed to *in vitro* assembly). Regarding the first challenge, it is noted that ssRNA is packaged at very high densities in icosahedral viruses (van der Schoot and Bruinsma, 2005; Aniagyei et al., 2008), which does not seem to leave much room for secondary structures. It may be possible to package small hairpins with the size of siRNAs but likely no longer dsRNA structures. An alternative would be the use of a dsRNA virus, for instance, CPV (Reoviridae), although a packaging system to incorporate foreign dsRNA segments would need to be developed. The existence of dsRNA structures in packaged VLPs is essential since no replication of the delivered RNA is expected and dsRNA as trigger of RNAi needs to be delivered as such. For the second challenge, efficient means must exist for packaging of the RNA in the capsids. Assembly of capsid proteins can be driven by specific signals in the RNA genomes but this is not always the case (Rao, 2006). In the case of cowpea chlorotic mottle virus (CCMV), packaging of RNAs of very different lengths (from 140 bp to 12 kb) could be achieved in the absence of specific packaging signals if the protein/RNA ratio was sufficiently high (Cadena-Nava et al., 2012). The major challenge therefore seems to be the synchronization of production of RNA and capsid proteins at the correct stoichiometry in the plant cells to generate functional VLPs at maximal efficiency.

Viral CPs can be used as delivery vehicles of toxins in the absence of formation of VLPs. For example, the RNA viruses of the family Luteoviridae replicate in plant hosts but are transmitted via a hemipteran vector in a persistent circulative non-replicative manner (Whitfield et al., 2014). After fusion of the CPs with an insect-specific peptide toxin and expression in *Arabidopsis* plants, the fusion proteins were found to cross the gut barrier into the hemocoel of the insect vector to deliver their aphicidal cargo (Bonning et al., 2014). CP-toxin fusions did not only target aphids that are the natural vectors but also non-vector aphids, suggesting their application as broad-spectrum aphicides (Bonning et al., 2014). Instead of fusion with a toxin, this property could be exploited by engineering CPs to transport dsRNA, for instance by fusion with a dsRNA binding domain.

The use of VLPs from plant viruses to deliver dsRNA to an insect is for several reasons advantageous over using the same viruses carrying their own genetic material. First of all, VLPs could combine safety for plant tissue and efficiency of cargo delivery to the insect. If produced in plant tissue, VLPs will not cause disease, in contrast to functional virions. Furthermore, they can form a specific dsRNA delivery method, since viral capsids are expected to interact with receptors in the gut epithelium to initiate entry in the insect's inner tissue layers (Smith and Helenius, 2004; Brault et al., 2007). Finally, packaging in VLPs is an effective way to protect dsRNA from degradation, similarly to the production of dsRNA in chloroplasts (Zhang J. et al., 2015). Thus, dsRNA is expected to be protected by the enclosing capsid proteins from nucleases and the dsRNA's integrity is maintained until it reaches its target (Galaway and Stockley, 2013; Itsathitphaisarn et al., 2016).

The well-known immunogenicity of VLPs is a property that has been harnessed to induce immune response against viruses when VLPs are used as vaccines (Chen and Lai, 2013). However, an issue that may arise by plant- or bacterially-produced VLPs applied against harmful insects is the unwelcome triggering of non-specific innate immune response in the plant that may lead to toxicity (Galaway and Stockley, 2013) or in the insect that may have an adverse (protective) effect against the VLP-dsRNA particles. Moreover, the efficiency of packaging of a foreign dsRNA is not guaranteed unless certain packaging characteristics for the virus that provides the CPs are specified.

## Safety aspects associated with the use of recombinant viruses for pest control

The evaluation of potential risks associated with recombinant viruses should include at least two levels of enquiry: the virus which is genetically modified and the fate of the recombinant material (summarized in Table 1). At the virus level, the specificity aspect has to be seriously considered if the recombinant virus is to be exploited for pest control in the field. For example, several reports have shown that most baculoviruses are not infectious toward predatory or beneficial insects outside of the order Lepidoptera, or toward othernon-targeted organisms. Hence, baculoviruses have unanimously been concluded in many studies as safe for use as pest-controlling agents (Kroemer et al., 2015). In contrast to baculoviruses, FHV is known to infect and replicate in insect cells of different insect orders (Dasgupta et al., 2003, 2007). Therefore, while FHV might make a good model for genetic modifications as viral delivery vehicles of dsRNA under confined laboratory experiments, it will pose potential risks if applied as a pest control agent.

**Table 1:** Safety aspects associated with the use of recombinant viruses for pest control.

	**Biosafety issues**	**Recommendation**
Virus specificity	Infection of non-target species	Selection of viruses with restricted host range. Host range of used virus strain (infectious clone) should be evaluated during risk assessment, by performing when necessary *in vitro* or *in vivo* infection studies (such as infection of the cells of non-target organisms) in addition to traditional PCR and sequencing methods
Transgene	(1) Transgene may present hazardous properties or change the vector properties	(1) Risk assessment should take into account the characteristics of the transgene (nature, stability, condition of expression), the construction/production process and the characteristics of the final recombinant vector (absence/presence of new properties compared to the virus backbone) and possible or known side effects related to the expression of the transgene
	(2) Non-target effects of dsRNA sequence	(2) Bioinformatic analyses of sequence complementarity between the pool of siRNAs and target genes in non-target species
Recombination	Establishment of a new vector with novel biological and genetic properties: (a) Genes that are interrupted or deleted in virus could be rescued during recombination (b) Transgene could be transferred to replication competent closely related viruses	Epidemiological data concerning the occurrence of natural closely related viruses in the area of administration should be analyzed to consider the necessity for *in vitro* or *in vivo* co-infection studies (between the recombinant vector and the potential natural closely related virus)

Besides the specificity of the recombinant virus to its target insect pest, special attention should also be put into the selection of the target gene sequence which will be exploited through the virus to control the insect pest. Non-target effects could arise if the RNAi targeted gene in the pest insect shares high sequence similarity to that of other insects, especially beneficial insects. The risk in this scenario could arise when beneficial insects, such as generalist predators, feed on the primary pest insects containing the virus-derived dsRNAs targeting a gene in the pest. As such, if high target gene sequence similarities exist between the pest and the beneficial insect, this could lead to gene silencing in the beneficial insect as well. However, for this to happen, the dsRNA will have to be stable in the gut conditions of the beneficial insect, to be taken up properly by the cells in the gut and in sufficient amounts to properly reduce the target gene transcript levels and finally the target gene will have to be as essential in the beneficial insect as in the primary pest insect.

Furthermore, the ecological consequences of the release of recombinant viruses have to be experimentally addressed in terms of the competitive characteristics of recombinant vs. the wild-type viruses, both in the greenhouse microcosm and in the field. Insertions into a virus genome could reduce the replication efficiency of the resulting recombinant virus, could affect the ability of the viral nucleic acid to be encapsidated properly, or could limit the ability of the recombinant virus to move from cell-to-cell or long distance within the host by affecting the folding of a native viral protein. However, considering that the virus-expressed insect-dsRNA is meant to accelerate the speed of kill of the target insect, and hence the recombinant virus itself in the process, this will imply that the modification does not confer any selective ecological advantage to the recombinant virus in comparison to the wild-type virus. In other words, compared with the wild-type virus, the recombinant virus is expected to show reduced fitness, resulting in lower concentration or even complete removal from the system.

Another possible concern associated with recombinant viruses is the potential of genetic recombination resulting in the foreign gene “jumping” from the recombinant virus to another organism. This could pose a risk if genes that are interrupted or deleted in a recombinant virus are either rescued during recombination or the transgene is transferred to replication competent closely related viruses. Recombination is a widespread phenomenon in viruses and can have a major impact on their evolution. Indeed, recombination has been associated with the expansion of viral host ranges, the emergence of new viruses, the alteration of transmission vector specificities, increases in virulence and pathogenesis, the modification of tissue tropisms, the evasion of host immunity, and the evolution of resistance to antivirals (Martin et al., 2011; Simon-Loriere and Holmes, 2011). Recombination seems to be highly frequent in some dsDNA viruses, where recombination is intimately linked to replication and DNA repair and can prevent the progressive accumulation of harmful mutations in their genomes. In contrast, recombination occurs at variable frequencies in (+)ssRNA viruses, with some families showing high rates (e.g. Picornaviridae), while others show only occasional (e.g. Flaviviridae) or nonexistent (e.g. Leviviridae) occurrence. The evolutionary reasons for the occurrence of recombination in RNA viruses are not clear. Since RNA viruses exhibit high mutation rates and large population sizes, it is more likely that these factors, rather than recombination, drive their evolutionary fate, as they regularly produce advantageous mutations and protect themselves from the accumulation of deleterious ones. Nevertheless, this does not exclude the possibility that natural selection can favor specific genotypes generated by recombination.

Several key factors limit or exclude the occurrence of genetic recombination between donor and recipient DNAs, including physical proximity (that is, localization in the same compartment within a single cell), degree of homology and similar modes of replication. However, if a recombinant virus pesticide is used long enough and at high concentrations in the field, it is expected that genetic recombination can eventually occur. In the field, as in laboratory conditions, such an occurrence is expected to be highest between highly homologous viruses (that is, the recombinant virus donor and wild-type virus recipient) that are infectious within the same host. The key question in terms of safety, however, is whether such recombination will result in an environmentally detrimental trait that will become fixed in the population. This will likely not be the case for recombinant viruses carrying a transgene for insect dsRNA expression, since a strong negative selection pressure arising from the rapid death of the target insect will lead to the recombinant virus being quickly outcompeted by the wild-type. Nevertheless, factors such as the homogeneity of the virus to be modified, the transgene and the possibility of recombination will have to be properly evaluated for each recombinant virus, prior to field application.

## Conclusions

Viruses, as the ultimate parasitic elements, have considerable advantages as delivery vectors of RNAi to eukaryotic cells. On one hand, they are uncannily specialized to enter and multiply in their host cells (efficiency); on the other hand, they usually have co-evolved with their hosts for long periods to maximize their reproduction (specificity). This review has analyzed in detail the prospects and challenges of the use of viruses as efficient and specific delivery agents of RNAi triggers (dsRNA). RNA viruses seem to be promising candidates since dsRNA is produced during the natural process of viral replication, while also DNA viruses have potential through expression of RNA hairpins. Imperative for the exploitation of viruses as delivery vectors is the availability of infectious clones or plasmid-based reverse genetics systems for the efficient manipulation of virus genomes. For different types of RNA and DNA viruses, such systems are available, as illustrated in Figures 1–4. Nevertheless, the success of viral delivery vectors for an RNAi effect faces several important challenges. Some viruses do not replicate efficiently in the host cells which results in persistent infections. During such persistent infections, the RNAi response may not be activated effectively and no silencing effects may be achieved. RNA viruses but also DNA viruses produce viral suppressors of gene silencing that may limit the RNAi effects that are intended. Thus, detailed knowledge of the viral infection process, most notably the initiation of viral gene expression and replication could provide valuable information with regard to the suitability of a particular virus to deliver RNAi efficiently.

Recombinant viruses could be produced in adequate production systems, such as insect cell lines or agro-infiltrated plant leaves, and then be purified and applied to the insect population, for instance through spraying. An alternative strategy would be the production of recombinant viruses in transgenic plants (under the condition that they do not cause disease or affect plant health); their presence in plant tissue will ensure their spread to the insects during feeding (Kumar et al., 2012). The use of recombinant viruses raises serious safety issues which need to be addressed. However, the plant and insect viruses that are proposed here do not cause disease in human or livestock and therefore benign regulation may be favored as is the case for baculoviruses.

VLPs may form a safer alternative to replicating viruses. In VLPs, non-replicating RNA with hairpin regions is encapsidated in viral particles that spontaneously assemble under the right conditions. Such VLPs can be regarded as sophisticated nanoparticles in which the capsid proteins function as effective and specific delivery determinants (Bonning et al., 2014). However, safety concerns remain also for VLPs and testing is required to investigate potential adverse effects against beneficial insects such as pollinators and predators/parasitoid wasps used as biological control agents. Although VLPs cannot replicate and potentially infect other hosts as a replicating virus might be able to, this advantage needs to be balanced against the costs and complexities of production and delivery, including timing. Whereas a replicating virus could potentially deliver RNAi over the life of a crop, VLPs are likely limited to effective protection of the leaves of the crop present when the VLPs are supplied, as there is no mechanism for spread as the crop grows, and new insect populations potentially migrate into the crop from elsewhere.

## Author contributions

AK and LS conceived the idea and designed the paper; AK, CT, and LS wrote the paper; CT prepared the figures of the paper; GS revised and contributed to improve the final version of the paper; all authors read and approved the final version of the manuscript.

### Conflict of interest statement

The authors declare that the research was conducted in the absence of any commercial or financial relationships that could be construed as a potential conflict of interest.
